# Patients’ and Health Care Professionals’ Expectations of Virtual Therapeutic Agents in Outpatient Aftercare: Qualitative Survey Study

**DOI:** 10.2196/59527

**Published:** 2025-03-26

**Authors:** Diana Immel, Bernhard Hilpert, Patricia Schwarz, Andreas Hein, Patrick Gebhard, Simon Barton, René Hurlemann

**Affiliations:** 1 Department of Psychiatry School of Medicine and Health Sciences Carl von Ossietzky Universität Oldenburg Oldenburg Germany; 2 Affective Computing Group Deutsches Forschungszentrum für Künstliche Intelligenz GmbH German Research Center for Artificial Intelligence Kaiserslautern Germany; 3 Leiden Institute of Advanced Computer Science Leiden University Snellius Gebouw Leiden The Netherlands; 4 Department of Computer Science Vrije Universiteit Amsterdam Amsterdam The Netherlands; 5 Assistance Systems and Medical Device Technology Department for Health Services Research, School of Medicine & Health Sciences Carl von Ossietzky Universität Oldenburg Oldenburg Germany

**Keywords:** socially interactive agent, e-mental health, mental illness, mental disorder, depression, major depressive disorder, suicide prevention, suicidal ideation, outpatient aftercare, artificial intelligence, virtual therapeutic assistant, public health, digital technology, digital intervention, digital health care

## Abstract

**Background:**

Depression is a serious mental health condition that can have a profound impact on the individual experiencing the disorder and those providing care. While psychotherapy and medication can be effective, there are gaps in current approaches, particularly in outpatient care. This phase is often associated with a high risk of relapse and readmission, and patients often report a lack of support. Socially interactive agents represent an innovative approach to the provision of assistance. Often powered by artificial intelligence, these virtual agents can interact socially and elicit humanlike emotions. In health care, they are used as virtual therapeutic assistants to fill gaps in outpatient aftercare.

**Objective:**

We aimed to explore the expectations of patients with depression and health care professionals by conducting a qualitative survey. Our analysis focused on research questions related to the appearance and role of the assistant, the assistant-patient interaction (time of interaction, skills and abilities of the assistant, and modes of interaction) and the therapist-assistant interaction.

**Methods:**

A 2-part qualitative study was conducted to explore the perspectives of the 2 groups (patients and care providers). In the first step, care providers (n=30) were recruited during a regional offline meeting. After a short presentation, they were given a link and were asked to complete a semistructured web-based questionnaire. Next, patients (n=20) were recruited from a clinic and were interviewed in a semistructured face-to-face interview.

**Results:**

The survey findings suggested that the assistant should be a multimodal communicator (voice, facial expressions, and gestures) and counteract negative self-evaluation. Most participants preferred a female assistant or wanted the option to choose the gender. In total, 24 (80%) health care professionals wanted a selectable option, while patients exhibited a marked preference for a female or diverse assistant. Regrading patient-assistant interaction, the assistant was seen as a proactive recipient of information, and the patient as a passive one. Gaps in aftercare could be filled by the unlimited availability of the assistant. However, patients should retain their autonomy to avoid dependency. The monitoring of health status was viewed positively by both groups. A biofeedback function was desired to detect early warning signs of disease. When appropriate to the situation, a sense of humor in the assistant was desirable. The desired skills of the assistant can be summarized as providing structure and emotional support, especially warmth and competence to build trust. Consistency was important for the caregiver to appear authentic. Regarding the assistant–care provider interaction, 3 key areas were identified: objective patient status measurement, emergency suicide prevention, and an information tool and decision support system for health care professionals.

**Conclusions:**

Overall, the survey conducted provides innovative guidelines for the development of virtual therapeutic assistants to fill the gaps in patient aftercare.

## Introduction

### Background

As stated by the World Health Organization, depressive disorders are among the most serious mental illnesses. They represent a serious loss of societal potential through premature mortality. It is estimated that 4.4% of the global population experiences depressive disorders. This is an increase of 18% compared to more than a decade ago [1]. The lifetime prevalence of depressive disorders is estimated to be between 16% and 20% [2]. Depressive disorders represent the most common mental health condition associated with suicide. It is estimated that 60% to 70% of individuals experience suicidal ideation during depressive episodes. The risk of suicide is 30 times higher than that observed in the general population. The mortality rate within the first year is approximately 3.4 times that of the general population. Furthermore, the probability of a depressive episode recurring within the first year is approximately 70%. In addition, approximately 21% of patients are readmitted to the hospital within 1 year of discharge [3]. The initial 6-month period following discharge is of particular significance with regards to the potential for relapse and suicide [4,5]. In contrast with the recommendations set forth in the German S3 guideline for unipolar depression, it is observed that 90% of patients diagnosed with major depression do not receive sufficient outpatient aftercare following psychiatric hospitalization [2]. This elucidates the elevated risk of relapse and suicide during the initial year [3]. Patients with restricted access to social, economic, or structural resources are particularly susceptible to these risks [2]. This conspicuous deficiency in outpatient care results in the forfeiture of potential therapeutic benefits that could otherwise be achieved through inpatient care. Conversely, enhancing outpatient care for patients with depression has been demonstrated to result in improved survival rates. In the context of this study, the term health care professionals refers to psychiatrists, psychotherapists, nurses and social workers who treat patients after they have been discharged from the hospital. The responsibility for patients with suicidal tendencies represents the aspect of working with depressed individuals that evokes the strongest emotional responses from this group of health care professionals and those who have previously cared for such patients. The experience of losing of a patient to suicide can have a substantial and lasting negative emotional impact. The risk of suicide among practitioners increases substantially following the suicide of a patient. In one study, 10% of participants, who were health care professionals, reported having suicidal thoughts themselves [6,7]. Thus, solutions that enhance the security of particularly susceptible patients, such as effective posthospital psychiatric follow-up, can consequently enhance the lives of patients and their families, as well as health care professionals, by minimizing occupational stress.

Before discharge, health care professionals need to assess the patients’ resilience in this regard and share responsibility. The decision-making process is often influenced by other factors, such as insurance coverage. Similarly, the factors that may lead to individual crisis situations are difficult to predict; therefore, postdischarge risk is also a professional burden for caregivers [8,9]. Although some patients continue to receive outpatient care after discharge, the sudden change in their situation poses several problems. One of the most prevalent problems seems to be the lack of a daily structure in time and tasks throughout the day, as provided by the hospital during inpatient care. Instead, patients are mostly responsible for their own daily structure, a task that many struggle with as their goal setting and persistence are not necessarily stable enough to overcome these hurdles. Conversely, in moments of self-doubt and struggle, they now have limited access to timely support. In the hospital, a safe environment with a wide range of support is available, especially in critical situations. However, this changes after discharge [10]. Outpatient psychiatric aftercare, when available, is limited and does not cover all critical situations that prevail in suicidal moments [11]. The limited number of health care professionals, together with time and emotional resource constraints and unclear responsibilities, pose a serious problem for patients. In addition, another source of support regarding social companionship, such as friends or family [12], may not always be available. More importantly, relatives are not professionally trained to deal with critical and potentially suicidal behavior. Some patients may even feel ashamed. In general, patients are left alone after discharge.

It appears that a gap exists in the system for outpatient care in critical situations, particularly with regards to the availability of health care professionals, the definition of responsibilities, and the lack of qualifications in many cases [2]. In the absence of in-person assistance, face-to-face support has declined and an increased demand for online support has been observed. This indicates a necessity for the development of secure, safe, and effective digital tools. Nevertheless, research indicates that internet-based cognitive behavioral therapies have a relatively modest impact on patients with suicidal tendencies [13]. Patients who continue to experience suicidal thoughts and behaviors also present a unique challenge for electronic mental health (e-mental health) services. Therefore, this group is explicitly excluded from e-mental health services [14]. A variety of interventions are available, including web-based psychoeducation, online counseling, SMS text messaging aftercare, email therapy, and computer-assisted cognitive behavioral therapy [15,16]. However, these interventions fail to address patients’ need for attachment, or for the formation of a social, empathic relationship. This has been described as the most important variable in determining the effectiveness of psychotherapy [17,18]. Consequently, the lack of a therapeutic relationship is a substantial criticism expressed by patients when evaluating internet-based cognitive behavioral therapy self-help methods [19].

It is thus evident that an intermediate professional solution is required to address the gap in care for patients with depression and suicidality, with the objective of providing support to both this susceptible patient population and their health care professionals. The solution should be readily accessible without overwhelming amounts of information, focus on depression-related cognitive and emotional impairments, and include suicide prevention. It should also be noted that using such solution, people in underserved rural or economically less developed regions could be better reached, overcoming socio-spatial barriers. Furthermore, the stress on the therapists in charge could be greatly reduced, thus contributing to staff stability in psychiatric institutions.

### Using Socially Interactive Agents for Outpatient Aftercare

Socially interactive agents (SIAs) can engage in verbal and nonverbal interactions as dialogue partners, exhibiting emotional and social capabilities and performing a diverse social support and monitoring functions [20-23], including provision of emotional support to users [24], monitoring of patient safety [25] and simulation of social skills to emulate social relationships, such as active listening, mimicry, gestures and emotion models [26-29]. Given the crucial role of social relationships in coping with psychological distress [30], there is the potential for SIAs to enhance technology-assisted outpatient care by emulating human interactions and tasks [31].

To date, SIAs have been used in a variety of settings as virtual coaches and assistants. Unified platforms for SIAs as coaches have been developed [32-34] and used for stress management purposes [35]. However, they have also been used in applied contexts as training partners for social skills [36] and empathy training [37]. In clinical research, initial approaches to the use of empathic conversational agents in the context of mental health have been proposed [38]. Furthermore, the use of relational agents has been demonstrated to have a positive impact on hospitalized patients with depressive disorders [39]. These findings suggest that these approaches may offer promising possibilities for the use of a virtual therapeutic assistant (VTA) as an assistant in a supportive, gap filling role in outpatient aftercare. It is unclear which settings a VTA could be used in and what role it should play. There is also a lack of clarity regarding the design of the interface for interaction with patients or health care professionals.

In SIAs, embodiment is of substantial consequence with regard to the manner in which they are perceived. While technology-mediated solutions can have a supportive effect, as previously outlined, communicating with a disembodied, faceless machine can impede positive outcomes, even when a human is on the other end. Conversely, embodiment in SIAs has been demonstrated to influence perceptions [40-42], increase trust [43,44], facilitate the formation of an emotional connection, and enhance overall relationship quality [45].

With regards to interpersonal interaction, a substantial body of research has examined timing, skills, and behavior of SIAs. It has been demonstrated that SIAs can evoke emotional responses that are pertinent to interpersonal interaction and attachment. The timing of SIAs behavior has been shown to be of critical importance in the formation of human-agent relationships [46,47]. In a job interview setting, SIAs were found to elicit feelings of shame to the same extent as human interviewers [48]. Moreover, analogous effects have been demonstrated with respect to trust [49] and rapport through backchanneling behavior [22,23,50], the elicitation of empathetic behavior in human physicians when dealing with virtual patients [51], and the elicitation of prosocial behavior in interpersonal interaction settings [52-54].

An embodied, affective SIA has the potential to facilitate novel avenues for relationship-based, technology-mediated outpatient aftercare. Nevertheless, its application to date has been confined to the context of scientific studies or hospitalization. The context of outpatient aftercare, which is particularly sensitive due to its applied nature, requires a meticulous and precise implementation of the VTA for it to have the intended supportive effect.

### Research Questions

#### Overview

This study presents a comprehensive analysis of the expectations held by both patients and health care professionals regarding the design of a proposed tool. The objective of the survey was to ascertain both expectations regarding the structural framework of how a VTA might interact with patients and regarding the interaction itself.

#### Role

The initial stage of the process entailed an evaluation of the potential integration of a VTA within the existing framework of outpatient care. It is necessary to establish the role that both groups anticipate a VTA will play in the context of outpatient care.

- Research question (RQ) 1: What is the role of a VTA in the context of outpatient care?

#### Embodiment

A substantial body of research and practical applications outlined previously indicates that the favorable outcomes of SIA interaction are contingent upon the manner of embodiment within the interaction interface.

- RQ 2: What are the embodiment preferences for a VTA?

#### Interaction Timing

The objective of this study was to address the expectations of patients and health care professionals regarding the interaction between a VTA and a patient. As no previous testing of this framework has been conducted, it is unclear what frequency and timing of interaction might prove beneficial for the patient.

- RQ 3: When should the VTA interact with the patient?

#### Interaction Abilities

Although a VTA offers numerous advantages to the patient, the specific interaction capabilities that the VTA should possess to be beneficial for treatment remain uncertain.

- RQ 4: What interaction capabilities should the VTA have to assist patients in their treatment?

#### Interaction Behavior

The following section will present the observed interaction behaviors and behaviors associated with the use of the aforementioned skills during the interaction.

- RQ 5: What interaction behaviors should the VTA use to facilitate patient treatment?

#### Interaction Between VTAs and Health Care Professionals

For the VTA to be considered an integral part of the treatment framework, it would be beneficial for it to provide useful information to the health care professionals themselves.

- RQ 6: How might the framework be designed in a way that would allow health care professionals at all levels (ie, psychiatrists, psychotherapists, nurses, and social workers) to benefit from the implementation of a VTA?

## Methods

### Overview

To investigate these questions, a 2-part qualitative study was conducted. The objective was to explore the perspectives of the 2 groups that were most directly involved (health care professionals and patients) and to assess their expectations of a VTA in the context of treatment. Initially, a group of health care professionals were queried about their expectations regarding the potential role of the assistant (RQ 1), assistant-patient interface (RQ 2), timing of the interaction (RQ 3), interaction skills (RQ 4), interaction behavior (RQ 5), and assistant–health care interaction (RQ 6). Following a comprehensive analysis of the responses, a qualitative interview was conducted with a group of patients undergoing treatment. This will provide a 360-degree perspective of the requirements that a socially interactive VTA should fulfill in a specific clinical setting to address the identified gap in outpatient aftercare. The methods and results are presented in parallel for the sake of comparability.

### Participants

In the initial phase of the study, data were gathered from a sample of health care professionals (n=30; 6 male and 24 female participants) employed in various outpatient settings within the field of outpatient care in social psychiatry in Oldenburg (Germany). All health care professionals participated voluntarily, which was ensured by providing them detailed information about the objectives and implementation of the study. No data were excluded. The age of the therapists ranged from 18 to 24 years (n=1), 25 to 34 years (n=13), 35 to 44 years (n=6), 45 to 54 years (n=4), 55 to 64 years (n=5), and ≥65 years (n=1), and their professions included psychiatrist (n=3), psychotherapist (n=10), social worker (n=15), psychiatric nurse (n=1), and physician (n=1).

The patient sample consisted of individuals (n=20; 11 male and 9 female participants) who were inpatients at the Karl-Jaspers Hospital in Oldenburg and were diagnosed with depression. All patients participated on a voluntary basis, which was ensured by providing detailed information about the objectives and implementation of the study. No data were excluded from the analysis. The age of the participants ranged from 19 to 56 years (mean 40.85, SD 12.46 years). Patients were administered the Beck Depression Inventory II (mean 24.3, SD 13.45; Beck et al [55]) to obtain an overview of their current depression status. To assess trust, the Yamagishi and Yamagishi General Trust Scale (mean 3.19, SD 0.72; [56]) was used, which comprises 7 statements, including “Most people are basically honest” with a rating scale from 1 (low) to 5 (high agreement), Cronbach α=0.83.

### Ethics Approval

Before data collection, approval was obtained from the Medical Ethics Committee of the University of Oldenburg (2022-005). Participants provided written informed consent to participate in this study. Data were anonymized. The participants received no compensation for this study.

### Procedure

At an informative meeting of health care professionals in our clinic, general information about the purpose of the study and details of the survey were delivered. Following the provision of a link via email, they were invited to complete a qualitative web-based questionnaire (implemented in Qualtrics) comprising a combination of multiple-choice and open questions (described in detail in subsequent sections).

The patient cohort was recruited through the clinical routine of their daily inpatient therapeutic examinations at the same psychiatric hospital. A semistructured qualitative interview was conducted in a confidential session. Following the introduction, a trained interviewer proceeded to elucidate the objective and structure of the forthcoming interview. It was emphasized that the responses provided by the participants would not be shared with any of the other patients and therapeutic staff.

### Materials

To ensure that all participants had the same level of knowledge about VTAs, a standardized description was first provided, which was adapted from the description set out by Hilpert et al [42,44]. The questionnaire was constructed in accordance with the 6 distinct expectations (corresponding to the 6 RQs) pertaining to a VTA in outpatient aftercare. The questions themselves were divided into 5 blocks, each addressing a specific area of inquiry: general expectations, specific situations, usefulness, communication and data handling, and demographics. The web-based questionnaire and the interview were comparably structured and comprised the same set of questions. However, the terminology and specific questions were tailored to align with the perspective of each respective group.

### Health Care Questionnaire

The questionnaire for health care professionals consisted of several sections. The initial section of the questionnaire addressed general inquiries pertaining to the health care professionals’ anticipated interface, capabilities, interaction behavior, and timing (eg, how and when to contact and how and when to support) of the VTA. In addition, it encompassed broader expectations related to the role of the VTA in the therapeutic process. Second, 2 particular scenarios in which patients may find themselves were delineated, accompanied by corresponding inquiries. In the initial scenario, health care professionals were instructed to envision a typical day in the life of an outpatient, beginning to end. Regarding competencies, the inquiries centered on the diagnostic abilities and subject matter that the VTA should possess to obtain a comprehensive understanding of the patient’s condition. The question pertaining to interaction timing addressed the optimal timing for the VTA to interact with the patient, while the questions concerning interaction behavior sought to ascertain the types of behaviors that health care professionals deemed beneficial. The second scenario pertains to an emergency situation that may arise during a day after discharge. The inquiry commenced with queries pertaining to the VTA’s capacity to procure external assistance for the patient and how the VTA could facilitate this. Subsequently, the participants were asked to indicate the circumstances under which they would interact with the VTA and to describe the helpful types of behavior. Third, the participants were requested to evaluate the utility of a VTA in relation to a series of therapeutic functions, including medication planning and crisis intervention. Fourth, we investigated their perceptions of their own interaction with the VTA, specifically regarding the type of information they would find beneficial if the VTA were to provide it to health care professionals involved in outpatient care. Finally, the participants were asked to provide demographic information.

### Patient Interview

The introductory information and briefing remained consistent throughout the process. As the interview with the patient sample was conducted after the analysis of the health care professionals responses, some of the questions were adapted based on the findings from this initial sample. This was done to elicit more detailed and nuanced responses and to avoid potentially vague responses. The overall structure of the questionnaire remained consistent. The questionnaire used in the interview encompassed the elements of RQ 1 to 5, excluding the interaction between the assistant and the therapist (as this did not impact the patients). The questionnaire was divided into 6 blocks: general expectations, assistant-patient-interaction, assistant-health care professional-interaction, feelings of the patient and demographics.

The interview commenced with a series of general inquiries pertaining to patients’ expectations of a VTA’s competencies, interaction style (including the modalities of contact, forms of support, and the VTA’s presence in the patient’s daily life), and a few more detailed questions concerning appearance. Subsequently, a description of 2 particular scenarios in which patients might find themselves was provided, accompanied by corresponding questions. Each specific situation was introduced with a subjective assessment of how the patients would feel in that particular situation. The questions were largely similar to those posed to the health care professionals, but the patients were also asked to provide an explanation for their responses. The section on usefulness was identical to that used with the health care professional sample.

In accordance with the recommendations for open research practices [57], all materials can be found on the OSF website [58].

### Data Analysis

To extract meaningful insights for each of the RQs in this qualitative dataset, a 4-step process based on grounded theory was used to analyze the responses given by both groups of interest [59]. This was executed following the guidelines described by Hansen and Grimshaw-Aagaard [60]. In the initial phase, the responses from each group of interest were transcribed (for the patient interview data) and organized by question in 2 distinct datasets, one for each group. Second, 3 independent raters classified the responses according to content and relevance to the RQs. Third, the raw data and extracted categories were compared for each group of interest (health care professionals and patients), identifying both similarities and differences between the 2 groups. Finally, this comparative dataset was classified to extract clusters and subclusters of explanatory value for each RQ.

## Results

This section presents the findings of the analysis of the health care professional and patient surveys. A joint analysis of both health care professional and patient responses is presented for each RQ.

### RQ 1: Role of the VTA

When queried about the role of the VTA, health care professionals primarily referenced the role of a “good friend.” It was emphasized that the VTA should be “designed in such a way that you enjoy the interaction, otherwise it’s probably not going to be used.” A similar argument was put forth with regard to the role of a social worker, which was described as “something between a good friend (feeling of closeness) and a social worker (knowledge about the disease and how to help).” Conversely, the explicit role of a VTA as a “therapist” was reported, with the addition that the VTA should be a figure with “distance to the family” or “not a substitute for family or friends,” stating that “these should be present in real life or newly built.” The VTA was recommended as an adjunct to existing therapy, with “more of an additional supportive role,” with an emphasis on supporting the patient “in phasing out therapy.” In a less role-specific description, the VTA was referred to as a “companion” or “extended inner support, more like a part of oneself that is currently too weak to provide self-support.” Health care professionals also described the VTA as a “coach” or “someone the patient can confide in, but who remains independent.” A more abstract idea was a “good spirit that no one else sees, who sees the positive and can sometimes be very direct but warm.”

Patients referenced the role of a therapist, “a known person,” as well as frequent references to a “good friend,” “being there for me like a best friend,” “asking questions about everyday life (instead of ‘psychological’ questions).” The wish for additional support in outpatient care is clear: “to work out goals and important topics with therapists, which the assistant knows about.”

In general, both groups anticipated a system that integrates expertise and represents a figure with whom they can establish a relationship. In conjunction with the primary recommendation of the therapist’s good friend, the role of a peer counselor would also be conceivable.

### RQ 2: Embodiment of the Assistant-Patient Contact

Regarding the topic of embodiment, we inquired about the participants’ expectations regarding general appearance characteristics of VTA. As previous research [40] has shown a clear pattern of human embodiment, we also asked about preferences for the assistant’s age and gender. The question regarding general appearance was posed in an open-ended format, with the objective of obtaining an unbiased report from the participants. Most of the patients (14/20, 70%) related their responses to the human appearance of the assistant. Some described nonhuman appearance and characteristics, to which age and gender do not apply. Regarding general appearance, the responses of both health care professionals and patients who endorsed a humanlike appearance were classified and summarized into 2 relevant clusters that play a role in the perception of the assistant: *physical appearance* and *expressiveness*.

In *physical appearance*, the health care professionals’ interview focused on 3 features: personal characteristics, clothing, and animation style. For physical characteristics, they tended to be the least risky, such as “medium height,” “normal weight,” “friendly face,” and “calm speech.” The same pattern was observed for clothing, as shown by responses such as “[the assistant] wears clothes in pleasant pastel shades, nothing too dark or too colorful.” The third feature could be defined as animation style, with health care professionals agreeing that the assistant should appear “as realistic as possible” with “no cartoonish features.” Regarding gender, 80% (24/30) of the health care professionals wanted a selectable option. The remaining participants recommended either a female assistant (3/30, 10%) or a diverse (3/30, 10%) assistant, while male gender was not explicitly recommended. Regarding age, a “middle-aged” assistant was recommended, as it should “exude life experience,” “not too young for older people.” Other recommendations were “age depending on the patient, maybe a bit older” or that the age should be “chosen by the patient.” For the *expressiveness* cluster, they focused on facial expressions and gestures that should be convincing enough for the assistant to be able to “respond to feelings and show empathy” as well as “natural movements.”

A different set of priorities for the *physical appearance* of a VTA was observed by the patients. While they wanted the assistant to have “an upper body and hands, but no legs,” it should “not have a perfect figure,” with the same mentioned for the face. Clothing was not mentioned. For animation style, they reported a tendency toward a more animated style “like in a computer game” or “like in science fiction movies.” However, in contrast to health care professionals, patients expressed a desire for familiarity, as the assistant should look like a known person, for example, the caregiver. Regarding gender, there was a clear and predominant preference of all participants for a female or diverse character. The reasons provided for this preference were “I am a woman myself,” “I can talk better to women,” “Navi, Siri are also women” and “it is easier to build trust.” In cases where patients did not explicitly prefer a female character, responses such as “voice is more important (soft, calm voice)” were given. It should be emphasized that none of the patients explicitly asked for a male embodiment for their VTA. Regarding the age of the VTA, “middle-aged,” “older,” but also contradictory answers, such as “between teenager and young adult” were provided. However, there was a tendency to favor a middle-aged figure to older figure. For *expressiveness*, it was desired that the assistant should be able to use facial expressions and gestures “so that emotions can also be clearly expressed” (clear facial expressions). Overall, they wanted “friendly” and “laughing” expressions.

Finally, there were some health care professionals who preferred a nonhuman appearance, citing concerns about “negative transference” or being “triggered” by a human figure. One participant illustrated:

I would like it if the character didn’t necessarily try to look like a human or even a therapist. I would be afraid that the difference would be too great and the character would look unbelievable or even ridiculous. I would therefore suggest that anthropomorphized, sympathetic avatars be created that patients could easily cast in a positive light.

Psychotherapist #35-44, male

Suggestions for this alternative, nonhuman appearance included a talking smiley instead of a real human face, or a “wise but humorous owl.” A few patients had different nonhuman suggestions, such as “blue water on the beach,” “self-selectable (robot, human, animal...),” “between emoji and human” and “can give itself a shape.”

These findings indicate that both groups anticipated an imperfect or neutral-looking but emotionally expressive human embodiment for their VTA, with a tendency to avoid male characters.

### RQ 3: Interaction Timing

The third RQ pertained to the timing of the interaction: when should the assistant interact with the patient? The joint analysis of the responses from both groups revealed 2 main clusters of situations in which the VTA could potentially interact with patients.

The first cluster was designated *situation-independent or proactive* and refers to behavior that does not depend on the socioemotional context of the patient at the time of interaction. Situation-independent or proactive interaction timing does not necessitate a request for assistance from the patient or the automatic recognition of an ongoing crisis situation by the assistant. This encompasses any form of interaction that could be proactively initiated by the assistant, including both scheduled contact and unscheduled check-in messages. For example, health care professionals proposed that the assistant could contact patients in the morning to “help start the day” and in the evening to “reflect on the day.” In between, at “agreed times” and “at random times.” Patients corroborated this assertion by requesting regular contact “maybe 1-2 times a day,” “daily contact,” “regularly in the morning,” “check in 3-4 times a day.”

The second cluster was designated *situation-dependent* and represents a complementary counterpart to the first. It includes all instances wherein the interaction is triggered by a response to the patient’s current therapeutic or socioemotional circumstances. This could manifest as the patient requesting contact, for example, to talk about a feeling at any time of the day, or even in crisis situations, such as a panic attack. It could also be triggered by monitoring the patient’s current stress indicators (eg, heart rate and lack of interaction) to detect an emergency situation and trigger a *situation-dependent* interaction in the assistant. Responses that were clustered as *situation-dependent* differed between health care professionals in the frequency with which this should occur. For example, patients mentioned “more often immediately after hospitalization” and that the assistant should contact them “on holidays/at weekends.” At this point, patients would like to be able to personalize the assistant’s presence according to their own needs. Patients would also like to be notified “as soon as he notices that I am not feeling well” or “if I have been inactive for a long time.”

Overall, health care professionals’ responses to the “when” question exhibited considerable variability. Regular or daily contact, fixed times, and contact when the patient is inactive were all represented to a similar extent. Although patients emphasized “unstructured” times when they expected to feel alone, there were no substantial differences between the responses of patients and health care professionals. These numerous variabilities may indicate the importance of an individual rhythm of contact.

### RQ 4: Interaction Abilities

This RQ was concerned with interaction skills: what competencies should the VTA possess to support patients in their treatment? The analysis revealed 2 main clusters of interaction skills that a VTA should possess in general: the capacity to furnish structural support and the capacity to furnish emotional support. Given the considerable overlap between the clusters extracted from the joint analysis of the responses of both groups of interest, it was determined that there was no additional benefit in reporting the responses of health care professionals and patients separately in this subsection.

The first cluster was defined as *structure-providing* capabilities, encompassing all the interaction capabilities of the VTA that facilitate structure for the patient regarding behavior (proposing action strategies or step-by-step plans for upcoming situations, monitoring the patient’s emotional state, and providing feedback) and time management (eg, structuring the day and reminder functions) to enable coping with daily challenges or tasks.

The second cluster was described as *emotional-supportive*, comprising skills that assist patients in managing their emotions in a relational context. This cluster comprised 3 subclusters: *supportive-trusting,* *supportive-motivational,* and *supportive-resource-promoting.* The capacity to be *supportive-trusting* enables the assistant to facilitate trusting conversations by being honest, authentic, interested, sensitive, nonintrusive, and reassuring. The capacity to be *supportive-motivational* enables the assistant to promote a positive attitude and increase self-esteem by acknowledging courage, praising achievements, and generally providing positive messages. The capacity to be supportive-resource-promoting enables the VTA to expand knowledge, increase self-efficacy and self-reflection, and develop and apply skills. Rather than giving instructions, this cluster emphasizes the promotion of patient autonomy through the development of self-management skills.

**Figure 1:**
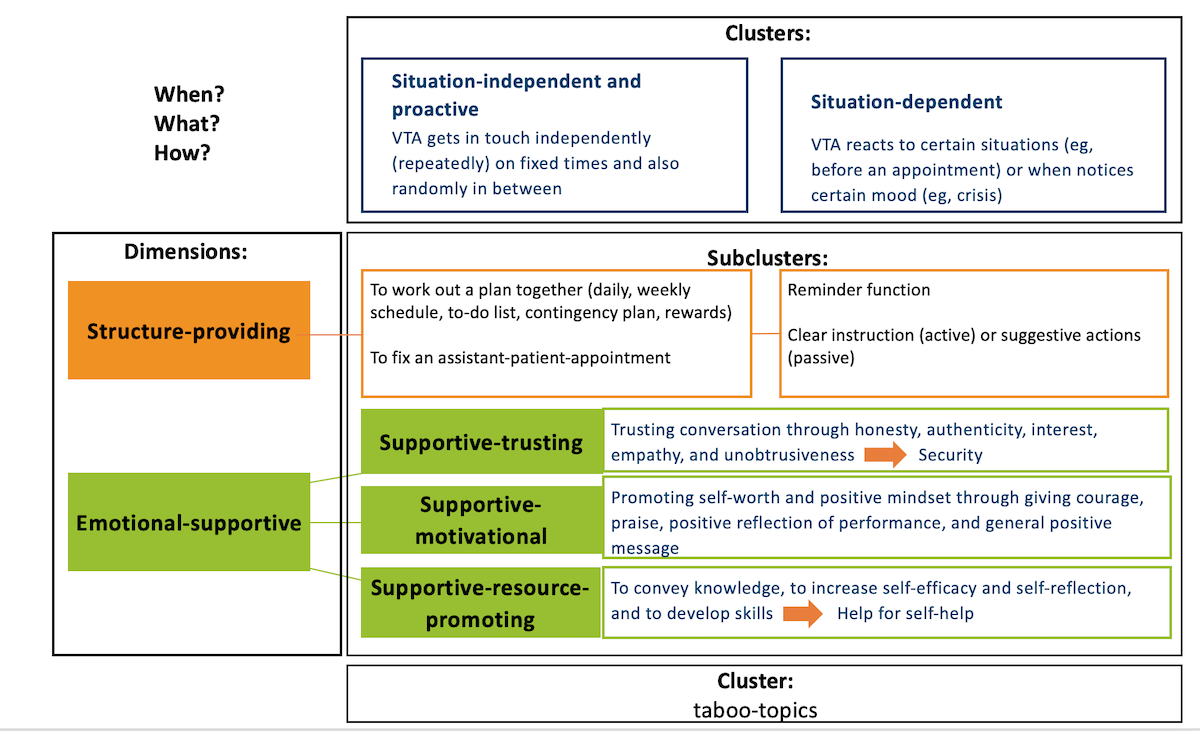
An overview of a 2D concept with clusters and subclusters in the interaction abilities of a virtual therapeutic assistant (VTA) in outpatient care. The definitions used to interpret the responses of participants (N=50, including health care professionals and patients with depression) in a semistructured qualitative study.

The results of RQs 3 and 4 provide a 2-dimensional conceptual space for interpreting participants’ responses. The first dimension characterizes the situational aspect of interaction timing and encompasses the clusters of *situation-independent or proactive* and *situation-dependent* expectations regarding the VTA. The second dimension pertains to the function of the interaction and encompasses the 2 clusters of expectations: one pertaining to *structure-providing* skills and the other to *emotional-supportive* skills. The *emotional-supportive* cluster may be further subdivided into 3 facets: *supportive-trusting, supportive-motivational,* and *supportive-resource-promoting*. This resulted in a total of 8 response subclusters, which can be used to categorize the interaction and the interactional behavior displayed. In addition, a further category of responses was identified as *taboo topics* representing a set of issues that should not be discussed. Figure 1 provides an overview of the dimensions and clusters with their subclusters, including individual definitions.

### RQ 5: Interaction Behavior

#### Overview

This RQ was concerned with interaction behaviors that the VTA should display to provide effective support to patients undergoing treatment. The responses are presented separately for each of the 8 subclusters of the conceptual space as described in the RQ 4: Interaction Abilities section. However, the questionnaire included a specific section of questions pertaining to an emergency situation or breakdown, the responses to which differed considerably from those provided in the general sections. Therefore, crisis behavior is reported in a separate section below.

Regarding *situation-independent* or *proactive structure-providing* behavior, health care professionals responded that the assistant could collaborate with the patient to develop a plan for the structure of the day, that is, “suggestions for organizing the day,” “checking off to-do lists” as well as “validating mood, suggesting daily activities.” Patients indicated a preference for regular contact, including “daily contact” and “welcoming words.” In addition, patients expressed a desire for a mood or habit tracker as well as daily advice.

For *situation-dependent structure-providing* behavior (ie, in situations of distress or instability), health care professionals recommended reminders, concrete instructions, or suggestions for action. These included “behavioral training,” “reminder of positive activities,” and “taking medication.” In addition, patients requested task reminders, eg, “appointments,” instructions on specific markers (eg, “sleep quality”) and “psychoeducation.”

Overall, health care professionals overwhelmingly stated that the assistant should remind patients of the strategies they have learned and provide them with positive support. Patients indicated a preference for the assistant to provide guidance and support through the provision of helpful suggestions and emotional support. There were no major differences between the responses of the 2 groups. However, health care professionals repeatedly emphasized the importance of providing positive patient-emancipating support, whereas patients increasingly indicated that they would prefer the assistant to inquire about their condition.

Regarding *situation-independent* or *proactive supportive-trusting* behavior, the health care professionals indicated that authenticity was a crucial element: “no good coaxing,” “moderate criticism,” and “avoid empty phrases.” Moreover, the VTA should monitor the patient’s emotional state and inquire about their well-being, while avoiding excessive intrusion (“don’t ask the same probing questions every day” and “don’t lecture, don’t put pressure”). The patients requested a personal connection and a relational memory to “refer to past episodes.” In addition, patients requested displays of empathy and humor, as well as affirmations of positivity and emotional support “as empathetic as possible” or “help to keep my head above water.”

In regard to *situation-dependent* *supportive-trusting* behavior, health care professionals emphasized the importance of honesty. A “perceived deterioration” in mood, “early warning signs” or “exceeding critical levels” should be communicated to the patient first. Moreover, the VTA should demonstrate interest by “listening when the patient tells you something on his/her own” and “keep the conversation going.” In addition, the assistant may proactively “engage the patient in conversation.” After specific incidents, the VTA may inquire about the patient’s experience by asking, “How did it go?” The assistant should demonstrate empathy and understanding, taking “negative or sad feelings seriously” and refraining from trivializing them. Unsolicited “funny” advice should be avoided. The patient should be queried as to whether they would prefer to discuss a “particular topic” in more detail or “at another time.” The patient should be “relieved” and “reassured” as much as possible. Patients in this cluster expressed a desire for honesty, interest, “being there for me like a best friend,” “empathy/understanding” and a sense of security “trusting that he will respond when I contact him.”

Overall, the responses were not markedly divergent, with both groups viewing the support worker as a primary source of assistance. Health care professionals had a strong focus on “avoiding harm” in anything the assistant would do, while patients’ responses reflected a fear of being left alone.

For the subsequent cluster, “situation-independent or proactive supportive-motivational behavior, health care professionals advised the presentation of positive or “thought-provoking” messages, “positive images,” and “music.” In addition, it was recommended that the daily routine be reflected upon by asking positive questions, such as “What are five things that have been good in the last week?” after which the VTA could summarize the successes to the patient. On the patient side, this cluster included promoting a positive attitude, “being supportive and encouraging,” self-esteem-boosting content, such as “responding to the patient’s stress and needs,” “not being annoying or condescending,” and general positive messages.

In the *situation-dependent* part of the *supportive-motivational* cluster, health care professionals mentioned “encouraging,” “promoting positive thoughts” and “praising” in relation to upcoming activities or occasions. In addition, patients mentioned “encouraging, praising” and “reminding of learned structures” to reflect positively on their performance.

Overall, the responses converged on the request for positive supportive behaviors, with the difference that health care professionals asked for proactive and patients for reactive behaviors.

In the *supportive-resource-promoting* cluster, health care professionals most frequently mentioned “psychoeducation” as a situation-independent behavior, with the addition that “reference to online forums should be avoided.” In addition, they suggested that calming techniques, such as “breathing exercises, mindfulness exercises” could be included. At this juncture, the transition to situation-dependent recommendations is fluid, as *supportive-resource-promoting* behaviors frequently prove effective for both types of timing. The health care professionals suggested that the assistant should help to “focus on the positive,” “address brooding thoughts,” and “motivate, show understanding.” As an example, for panic attacks, breathing exercises could be used to help. For behaviors that are exclusively mentioned as situation-dependent, health care professionals mentioned providing explanations, for example, “in bad phases, the changeability of feelings and mood phases” could be explained with reference to individual examples from the patient’s past. As this type of self-help promotion was considered particularly important by the health care professionals, an exemplary list is provided in Textbox 1.

**Examples of how a virtual therapeutic assistants can be used to promote self-help for patients with depression in the outpatient setting, which is considered particularly important by health care professionals (n=30) in a semistructured qualitative study.**

**Examples for self-help promotion:**

- “Where could your bad mood come from?”
- “What has helped with low mood so far?”
- “What self-stabilization options do you have?”
- “Who could you call?”

**Situation-independent reminders should also include the following:**

- Knowing about or instructing, if not already available, the patient’s skills, strategies, and stability factors
- Methods for dealing with circles of thought and rumination
- Defining emergency contacts together (making them quickly accessible)
- Informing about outpatient help offers (self-help groups, outpatient treatment, telephone counseling, specialists, and social psychiatric service)
- Building up activities (eg, maintaining social contacts) and providing inspiration for activities
- Supporting eating behavior (only if desired) and sleep hygiene
- Homework support

Patients in the *supportive-resource-promoting* cluster requested specific forms of assistance and guidance, including “offering autogenic training,” psychoeducation, and dialogues—enabling conversations between the assistant and the patient as a situation-independent behavior. However, monologues and input from the assistant, such as “nice topics” and “providing facts and clear lines” were also requested. In the situational dimension, patients expressed a desire for biofeedback, skills and competencies—showing ways to solve problems,” relational memory—“referring to past episodes,” and active dialogue—“talking to me about what is bothering me.”

Overall, regarding *emotional-supportive* dimension and its 3 subclusters, practitioners indicated that they would use specific strategies and interactional behaviors to facilitate conversations through honesty, authenticity, interest, empathy, and unobtrusiveness to build trust and convey safety. To motivate, positive self-esteem and minimum standards should be achieved through encouragement, praise, positive reflection on achievements, and general positive messages. The objective is to provide knowledge, enhance self-efficacy and self-reflection, and cultivate skills. Patients tended to mention more functional characteristics at this point, whereas practitioners were more likely to cite the assistant’s capacity for empathic understanding.

A further category of responses was identified, namely a cluster of *taboo* *topics* that should not be discussed “because the questions might trigger and the patient would be left alone with negative feelings.” These included suicidal thoughts, weight gain, and possible crises that could not be detected, “risk of not being able to detect this in an individual way, or inadequate response to suicidality.” Nevertheless, some health care professionals advocated for the inclusion of all topics and the elimination of taboos, stating “I think there should be no taboos. Social interaction should play a big role, sexuality can also be mentioned.” Additional responses on this page indicated that: “In general, the character should address many topics so that the patient thinks about his condition” and has to “show his colors.”

A similar list of topics was identified in the patient responses. Topics that are deemed irrelevant or potentially “thought triggering” should be excluded from discussion. Furthermore, certain intimate topics should be avoided, as respondents indicated: “For many topics I would need a person.”

In general, when queried about the appropriate conduct of the assistant, both respondents concurred that the assistant should refrain from exerting undue pressure or authority and should avoid engaging in repetitive or critical interactions. There is a distinction in the manner of presentation, with patients describing their preference for an assistant who is not “annoying,” “pointing out mistakes,” “using empty phrases,” and “scolding.” Health care professionals have indicated that the assistant should not be “pushy,” “lecturing,” “trivializing,” and “admonishing.”

#### Crisis Behavior

When queried as to how the assistant could facilitate a secure treatment environment during a crisis, health care professionals posited that the assistant could facilitate the establishment of contacts (personal and professional), “motivate [the] patient to turn to friends/family/therapists,” continue communication by “maintaining dialogue with the person.” Additional strategies included providing reassurance to the patient “Keep calm! Remind them of what has been agreed in advance,” assuring them of support, “reassuring them” by “announcing supportive next steps and being as transparent as possible” and providing positive support: “Be transparent, do not deny that it is only digital support, only do what has been discussed with the patient beforehand and what they have agreed to.”

A noteworthy response tendency emerged from the analysis of the practitioner questionnaire: “motivate the patient to seek help.” In response to this, we explicitly asked the patients how the assistant could most likely motivate them to seek help. The respondents indicated that the assistant should refrain from calling for help prematurely and should instead afford the opportunity to ask for help first. However, half of the respondents indicated that the assistant should directly request assistance, indicating a preference for contacting the therapists rather than friends or family members. In the context of a crisis situation, patients also expressed a desire for the assistant to maintain objectivity, to be “factual/informative about the situation (what benefit of seeking help)” and to “communicate mood clearly (visually).” Patients also wanted the assistant to be authoritative: “Just say: It’s time to get help” or “I’m convinced that help is appropriate now (as an announcement).” However, they also wanted to be motivated by the assistant’s reassuring behavior “by using a reassuring explanatory address (not ‘You have to do it now,’ but ‘Maybe you should...’).” In addition, the concept of a “panic button” was proposed, along with the display of assistance contacts, the presentation of support alternatives, and the provision of psychoeducation.

Overall, a wide range of behavioral options for the crisis situation was desired, especially by patients. In contrast to the patients, authoritarian behavior was not desired by the health care professionals. However, there was a tendency toward a preference for the system to be able to call for help without the patient having to give consent in a crisis, showing affinity toward decision-making authority. This may reflect the need for patients in crisis to be able to relinquish responsibility or to find guidance in the often-difficult decision-making process about whether and when a call for help is appropriate and necessary.

### RQ 6: Interaction Between VTA and Health Care Professionals

This RQ aimed to investigate the relationship between the VTA and the health care professionals themselves. What modifications could be made to the framework to ensure that health care professionals at all levels could benefit from the implementation of a VTA? When queried on this matter, the health care professionals indicated that this should be done at the patient’s request, if the patient is able to provide consent. It is recommended that a preestablished “limit” be set regarding when the practitioner should be contacted. Moreover, the assistant should “obtain the patient’s permission beforehand.” In such instances, the assistant is permitted to “report suicidal thoughts, progressive deterioration of mood, or documented deterioration of mood over a period of 1-2 weeks,” “if a constructive discussion is not possible.” A further cluster of considerations pertains to instances of potential danger to self and others, “if the virtual character cannot assess the further danger with certainty,” “with threat or lack of dissociation of self—or harm to others,” or with “report of suicidal thoughts (even once).” In particular, in the absence of clear dissociation of suicidal thoughts, it was proposed that the assistant could potentially dial the emergency number in the event of inactivity and a lack of response from the patient for a predefined period, in the case of an unannounced exit from the program, or in the event of inactivity for a period exceeding 48 hours. The final 2 clusters were identified as imminent when the “SOS button” was pressed or in the case of “external stressful events and crisis situations.” At this juncture, the patients casted their votes on the question of whether the therapist should be informed without the patient’s consent. The votes were one in favor and one against. A single individual posited that the assistant should be permitted to establish contact without delay, if the patient has consented. In total, 3 patients indicated a preference for multiple inquiries before action. This reinforces the necessity for individualized approaches and step-by-step plans that can be triggered according to the circumstances.

## Discussion

### Principal Findings

In the first part of the research findings, we examined how a VTA could be used as part of ongoing outpatient care. This includes the role (RQ1) of a VTA, particularly in the potential of filling the treatment gap, after discharge from the hospital. The respondents anticipated a system that would integrate expertise and facilitate the establishment of relationships between individuals. In conjunction with a primary recommendation from a trusted confidant of the therapist, the role of a peer counselor was also considered. Results on VTA embodiment (RQ2) indicated the need for such a counselor and provided practical implications, such as an imperfect or neutral-looking but emotionally expressive human embodiment, with a tendency to avoid male characters. In the second part of the findings, we evaluated patient and health care professionals’ expectations regarding the interaction between a VTA and a patient. The results on interaction timing (RQ3) were considerable variables, reflecting the importance of an individual rhythm. The results obtained were delineated into 2 main clusters: *situation-independent* and *situation-dependent*. Regarding interaction abilities (RQ4) that the VTA should offer to support patients during treatment, 2 main clusters were also identified. *Structure-providing* included behavior for structuring and time management. *Emotional-supportive* included 3 subclusters *supportive-trusting,* *supportive-motivational*, and *supportive-resource-promoting.* The results regarding interactive behavior (RQ5) are presented separately for each of the subclusters identified in RQ3 and RQ4 and include explicit, competence-related, or emotion-stabilizing behavioral instructions. In addition, another cluster was identified as *taboo topics*, which includes content that should not be discussed.

The results pertaining to the interface between the assistant and the treating professionals (RQ6) are particularly relevant in determining the content that should be shared, the frequency, the timing, and patients consent.

### Role

Expectations of the role of the assistant strongly reflect the treatment gap—both groups recognized the potential of a VTA as a supportive anchor following hospital discharge, not constrained by availability of health care professionals, but still equipped with professional techniques to navigate challenging circumstances (that relatives may not possess), while simultaneously serving as a projection screen for personal attachment. This leads us to conclude that a VTA has the potential to address the treatment gap, at least in part, and thus enhance outpatient aftercare.

### Embodiment

This survey replicates previous findings [40] showing the need for an embodiment of the VTA and provides further insight into the practical implementation of this perspective. At first glance, the patients’ expressed desire for an imperfect figure that could be interpreted as an indication of the “uncanny Valley effect” [61]. This phenomenon describes the abrupt decline in the acceptance of robots when they become too similar to humans. Nevertheless, the statements explicitly refer to aesthetic features, such as the (imperfect) figure and suggest that the assistant should not evoke negative social comparisons or negative self-evaluations, which can be a symptom in patients with depression. In this context, patients express fears of doubting their own self-esteem [62]. A common clinical presentation among individuals with depressive disorders is a perceived lack of self-esteem [63]. It would be beneficial to consider whether the VTA could be helpful in demonstrating how to make self-evaluation more positive, to show solution strategies. Notable examples include the chat robot Webot, which uses self-learning mechanisms through the presentation of short narratives about itself [64], or the home robot Jibo, which uses positive psychology techniques to assist individuals through the day [65].

A striking tendency in our data was the recommendation regarding the gender of the virtual assistant. Here, neither health care professionals nor patients explicitly recommended a male character; rather, a female character or a character with selectable gender was the preferred option. In other words, when explicitly asked to select a gender for a VTA, both health care professionals and patients avoided choosing a male character. Considering the highly sensitive target group of inpatient depressive patients on which our data were based, this recommendation suggests that the figure of the assistant should not convey any sense of threat. Other indications from the results regarding appearance included a “soft, calm voice” and “pleasant pastel shades, nothing too dark...” It can be concluded that men are perceived as more threatening according to the specific needs of the target group with depression. Statistical evidence indicates that instances of domestic violence against males and same-sex domestic violence are perceived as less threatening to society than instances of domestic male-on-female violence [66]. Research indicates that domestic violence has a profound impact on individuals and families and it is a substantial risk factor for mental illness [67]. In light of the observed tendency to perceive women as more empathetic, a phenomenon that has been documented in previous research on VTAs [68], it is plausible that the gender recommendation may have a dual impact here. It can be posited that both patients and health care professionals subconsciously had a preference for a female figure due to the aforementioned associations and the underlying motive to establish trust and security. Conversely, research on stereotypes in the form of dominance and gender in the VTA indicates that male-associated behavior in female VTAs that does not align with the stereotype is met with rejection [41]. This indicates that it is crucial for gender-related conduct to align with the character’s physical appearance. With respect to the specific target groups under consideration in this study, the impact of human-agent interaction in this regard should be examined, particularly in light of the notable gender-related outcomes observed here. Furthermore, these findings could be extended to other applications of SIAs where gender is a pivotal factor.

Some patients expressed a desire for the VTA to be modeled on their personal therapist. In light of the potential for technical advancement to enable the realistic modeling of avatars based on specific individuals [69], this option may be regarded as a realistic prospect. The therapeutic advantages (eg, availability of assistance) or disadvantages (eg, increased dependence on the therapist) would be highly specific to the particular therapeutic function or therapeutic relationship in question. As robots and intelligent systems begin to blur the boundaries between physical and virtual reality, there is an increased risk of negative emotions and thoughts being transferred to an assistant that has been modeled on someone from the patient’s life. It remains to be seen whether intelligent systems will develop the capacity to counteract such effects in the future. This would require careful research [70]. However, the desire to recreate a known person as their VTA reflects patients’ fear of being left alone and without trusted support after leaving the hospital. This was a common theme throughout this survey.

Regarding external expressiveness, patients and health care professionals indicated that the assistant should be capable of communicating through facial expressions and gestures. It is recommended that the face, upper body, arms, and hands should be visible. In addition to voice and content, body language is also of importance. This indicates that the affective functions of the assistant should be multimodal to be able to act emphatically. In crisis situations, patients may engage with such a system with their undivided attention. In contrast to other contextual studies, such as those examining a driving assistant where users have only a divided attention available for interaction with the assistant, it is desired for VTAs to be less distracting (only one visible head; [41). In addition, it is demonstrated that participants want to allocate more attention to the assistant and want it to be expressive and empathetic. The research on nonverbal synchrony in therapeutic sessions indicates that high levels of nonverbal synchrony are associated with a higher quality of the therapeutic relationship and greater symptom reduction. Consequently, the design of an assistant capable of nonverbal synchrony with the patient serves as an additional indicator of therapeutic efficacy [71]. Inferentially, through an assumption of an outward appearance with corresponding expressiveness, participants in both survey groups expressed a desire for this function. However, it is of particular importance, particularly in the context of mental health, to design expressive VTA behavior in accordance with the specific requirements of the health care target group [72].

In conclusion, the findings indicate that embodiment is a substantial factor, but its implementation must be approached with caution to ensure a beneficial impact.

### Timing

In response to the question of timing, the 2 main clusters were identified as *situation-independent* and *situation-dependent*. The results of the *situation-independent* cluster may also be summarized as proactive behavior on the part of the assistant. Proactive behavior on the part of the patient was not mentioned by any of the respondents. In general, this demonstrates that the assistant is regarded as an activating medium and the patients are perceived as passive recipients of data by the therapists and by themselves, except in situations where requests are situation related. Here, the particular requirements of the specific depressive group are evident. This indicates that the initial 6-month period following discharge is a critical period for the risk of relapse and suicide [4]. The reintegration into to the home environment frequently results in incomplete remission, and the persistence of symptoms further complicates the ability to independently coordinate the various stakeholders in sociopsychiatric aftercare. The accompanying psychosocial restrictions, such as a lack of daily structure or cognitive limitations, make the necessity for a situation-independent, activating figure apparent. The almost unlimited availability of activating resources of such a system appears to be of great functional advantage at this point. Therefore, the unconstrained accessibility of activating resources within an attachment-oriented system gives rise to concerns that are not seen in the natural boundaries of human-human interactions. In light of the aforementioned limitations, a situation-independent activating assistant appears to be a logical solution for patients with depression during aftercare. However, there is a risk that unrestricted availability of the attachment-oriented assistant may result in dependency among patients. For example, a previous study [73] on attachment-oriented robots demonstrated that a system that provides emotional attachment in addition to factual competencies and addresses loneliness may potentially foster dependency in patients. It is yet to be determined whether a system designed to counteract this can be developed in a way that incorporates factual competencies at all levels into a training mode that is inherently geared toward resource building, thereby creating space for development and independence.

The frequency-related results, that is, the question of when the assistant and patient should interact, demonstrate the necessity for a more individualized system. The need for individualization can be regulated in 5 different ways: at the request of the patient, according to content, according to time, when the patient’s condition changes and before outpatient therapy or appointments.

Regarding frequency of interactions, these may occur daily at designated times, on a weekly or monthly basis, or at other intervals. Interactions surrounding particular events may also be structured temporally in accordance with scheduled appointments. Human-interaction therapy sessions in a social psychiatric context are typically conducted at varying frequencies. At the outset of a treatment agreement, a potential frequency is typically established, contingent on the patient’s needs and the therapist’s availability. Research has shown that participants, especially those with lower levels of education, preferred a VTA to a human after discharge from hospital because they could control the pace of information [74]. Furthermore, the frequency of therapy appointments tends to increase gradually, contingent upon the efficacy of the therapy and the availability of approved cost coverage. In this context, the frequency of therapy has an impact on the success of therapy. Consequently, researchers advise therapists to treat patients as often as possible, particularly during the initial phase of therapy [75]. At this juncture, a virtual system can bridge gaps in care due to its unlimited availability and supplementary therapies that are more limited in scope. Conversely, it is of the utmost importance to prevent the patient from becoming dependent on the therapist or on a digital system. It is therefore imperative that a digital system, which is capable of maintaining unlimited availability, simultaneously instills a sense of autonomy and independence to prevent dependency. Should the patient wish to interact, this would necessitate the individual activation of the system by the patient. Content-dependent interactions refer to the dialogue or content that the patient shares with the system, either verbally or by answering questions. The individualization of the content of the dialogue represents a key aspect of this process. Interactions that occur when the patient’s emotional state changes can be proactively initiated by the patient. It is similarly conceivable that this could form the basis of a digital phenotyping monitoring system. The concept of being “seen” by a system that responds to crises, provides assistance, or alerts to early warning symptoms was generally viewed positively. New interventions that take advantage of the quantification of clinical markers through condition monitoring can contribute to clinical improvement [76,77]. Our results indicate user acceptance of such monitoring at this point, as well as needs and preferences.

### Interaction Abilities

Regarding the interaction skills that the assistant should be able to offer to provide support to patients undergoing treatment, 2 broad clusters were identified. On the one hand, the skills that could be classified as structured assistance were designated as the *structure-providing* cluster. In contrast, the skills that could be assigned to emotional support were classified as the *emotional-supportive* cluster. Both of these main clusters could be defined in a cross-dimensional manner, thereby allowing for the identification of a higher-order meaning. In this context, we focused on 2 sociocognitive dimensions that are particularly relevant for the description of individuals: warmth and competence [78,79]. These 2 variables have been identified by Fiske et al [78] as the 2 most important factors by which people evaluate encounters. The character traits that individuals form about others when they spontaneously gather impressions can be summarized in these 2 basic dimensions [80]. The dimension of warmth is defined as a set of traits that are perceived to indicate intentions of friendliness, helpfulness, sincerity, trustworthiness, and morality. In contrast, the competence dimension traits are associated with perceptions of abilities, including intelligence, skill, creativity, and efficacy [78]. Our data point to these sociocognitive dimensions within the 2 main clusters.

In addition, the data delineates the interaction skills, which include the virtual assistant’s emotions, personality, and social skills. In conjunction with the character’s appearance, these skills influence the user’s assessment of the character’s believability. The term “believability” in the context of VTAs is defined as the capacity to be perceived as a genuine entity [81]. For this to occur, the character must act in a manner that is consistent with its stated goals, personality, and state of mind [82]. In this context, consistency is defined as the coherence between the characters’ verbal and nonverbal behaviors as well as its physical appearance. In particular, our data substantiate the hypothesis that users of virtual assistants within the subcluster *supportive-trusting* desire consistency in the assistant’s behavior. This desire was described as “authentic” as well as socially interactive and competent in the sense that the assistant’s emotional expressions and statements should be appropriate to the situation. It is therefore evident that the VTA must be capable of modifying its social conduct in accordance with the circumstances to appear credible and pertinent. Our findings corroborate those of previous research, which have highlighted the significance of believability in virtual assistants [81]. In addition, our data indicate that believability can be classified as a subcluster of the warmth dimension, and can be seen as a subconstruct of the 2 sociocognitive dimensions of warmth and competence.

### Interaction Behavior

In contrast to human therapeutic interactions, which always entail an exchange about a momentary, subjective state and require the patient to disclose personal content, a digital monitoring system maintains a pervasive and comprehensive impression that may not necessitate an active decision by the patient to communicate. At this juncture, users of a digital monitoring system are disclosing a considerable amount of personal information by sharing data that is not actively shared, thereby relinquishing both responsibility and control over the sharing process. The data indicates that, regarding the variety of tools and forms of intervention, there is no substantial bias toward data sharing among the 2 groups of respondents. The primary objective is to identify a figure who can effectively combine competence with warmth, thereby fostering the most robust possible trust between the patient and the figure. As evidenced by psychotherapy research, trust and shame play a major impact in the efficacy of therapeutic interventions [83,84]. Assisting patients in modifying their behaviors also necessitates transparency regarding their shortcomings. Concurrently, individuals divulge a considerably more intimate array of information to computers than to humans [85,86]. Despite the well-established fact that data is identifiable, indelible, and, in certain circumstances, obtainable [87], computers do not elicit the same social inhibitions as humans. In the context of human–computer interaction, particularly in the context of digital assistants, it is pertinent to inquire into the underlying mechanisms that facilitate this phenomenon. Against this background, it seems plausible that crucial psychometric inquiries in psychiatric diagnostics may be more effectively elucidated through human-machine interaction. It is beyond dispute that the protection of patient data must be accorded with the highest priority. In its social function, shame serves as a reflection of the affected person’s behavior and has developmental potential. Conversely, shame entails the participation of at least 2 individuals, thereby indicating that the observed transgression was inadvertent. In this way, shame functions as a feedback mechanism, protecting against extremes or loss of control and leading to inclusive behavior in interaction partners. In social interactions, the successful overcoming of shame can facilitate the formation of attachment on both sides. However, existential shame, which is defined as the perception of oneself as generally flawed, can result in social withdrawal and, ultimately, the onset of depressive symptomatology [88,89]. It is, therefore, possible to hope that deficiencies in care in human-machine interaction can also be addressed at the level of communication. A solution to social psychiatric diagnostics that is free from shame can help to prevent crises, particularly in situations of critical transition, such as after hospitalization. At the same time, it should be noted that a shame-free interaction with a computer will not replace the challenges that people experience in social encounters.

### Assistant-Health Care Professional Interaction

This final inquiry relates to the interface between the assistant and the health care professionals, that is, modifications to the framework to ensure that health care professionals benefit from the implementation of a VTA. Moreover, this could be regarded as a control function to guarantee that the assistant does not unintentionally impede the ongoing treatment.

The results from all cluster dimensions demonstrate substantial overlap across the full spectrum of social psychiatric care, which raises the question of how such a support system could potentially transform the landscape of social psychiatric care. For example, it could result in a shift in social values within the domain of social psychiatry or contribute to an increased reliance on digital technologies for the provision of care. Accordingly, the societal impact of an intelligent, attachment-oriented assistance system on both human-human and human-robot relationships must be explored and designed to be participatory at all levels of use [90]. Evaluations need to take place at all levels of development in this process, so that all targeted professional groups, as well as patient advocacy organizations, can provide input [76]. In particular, possible short- and long-term effects on the individual patient’s identity, freedom of choice, and self-perception need to be explored.

Two potential outcomes emerge at the interaction interface between therapist and assistant, as evidenced by the overall results. On the one hand, there is the possibility of receiving information about the patient’s health status via a ubiquitous computer, which the patient would be disinclined to share with the perhaps new, still unknown therapist for reasons already discussed. The primary reasons for this were insufficient time or a lack of confidence on the part of the patient. Conversely, the alarm system serves to alert the therapist or the rescue system in the event of indications of suicidal ideation. These 2 points present the potential for addressing existing care and enhancing the safety of patient follow-up for those with depression [91]. Concurrently, the frequently arduous period of follow-up care will be less onerous for treatment providers. The enhanced objectivity of the assessment of patients with depression who are to be monitored provides a basis for decision-making in the context of treatment.

It would be beneficial to ascertain which groups of patients are particularly at risk of relapse following hospitalization. Those from socioeconomically disadvantaged backgrounds are frequently inadequately served. Psychiatric care is underrepresented in rural, economically underdeveloped regions [1]. Furthermore, the risk of relapse is elevated in individuals who are young at the onset of illness, female, older, have somatic comorbidity, are single, and have a general lack of psychosocial support determine a high susceptibility to relapse [2]. During the course of the pandemic, the rise in the incidence of mental health disorders occurred concurrently with substantial disruptions to the provision of mental health services [92]. A deficiency in the provision of essential health services was observed, including those pertaining to mental health and suicide prevention, as reported by the World Health Organization. While the situation showed some improvement by the end of the pandemic, it still demonstrates the vulnerability of a health care system to unforeseen crises. Health care professionals caring for these susceptible people could benefit professionally from a VTA system that helps to make outpatient care safer. The effect sizes, considering the different groups of people affected, remain an open RQ.

### Limitations and Directions for Future Research

This study has several limitations. First, the recruitment and questioning of the 2 groups of participants was carried out in different ways, with the questioning being adapted both to the specific groups and to the specific situations. This could have led to distortion. Second, the results showed that the answer option “diverse” to the gender question was misunderstood. Future studies should pay attention to clearer formulation. In addition, psychometric data were used solely for descriptive purposes, despite the potential for exploring the impact of individual factors on outcomes, a direction that merits attention in future research. The study’s scope is further limited by its inclusion of health care professionals and patients from Oldenburg, Germany, precluding the generalizability of its results. To enhance the relevance and external validity of the findings, subsequent studies should aim to encompass more representative samples of the general population. Furthermore, this study exclusively examined participants’ attitudes toward an SIA, neglecting to investigate its potential effects on patients. Consequently, further research is necessary to investigate the impact of such a system and to ensure the benefits for those affected at all levels of the system.

### Conclusions

Main conclusions can be derived from the study related to the role of VTAs, their embodiment, the timing of interaction, interaction skills and behavior, and interaction between the assistant and health professionals.

#### Role

The VTA was identified as a valuable supplementary component within the outpatient aftercare system. The VTA is able to address deficiencies in care due to its extensive repertoire of therapeutic skills and its capacity to establish a rapport with patients where social workers, therapists, and family members are unable to do so.

#### Embodiment

A multimodal function for empathic communication (ie, voice, facial expressions, and gestures) is expected. Patients may be more likely to engage with an assistant who appears imperfect, as this may help to prevent negative self-evaluation, particularly in patients with depression who lack self-esteem. The assistant may be able to assist in the development of solution strategies to enhance a more positive self-image. The gender of the assistant should be either female or selectable, but not male. This preference is thought to be based on negative associations with the male gender, such as domestic violence, and the perception of the female gender as more empathetic.

#### Timing of Interaction

The findings indicated that both patients and health care professionals anticipate the assistant to engage in proactive behavior. Therefore, the assistant is regarded as an activator of information, whereas patients are viewed as passive recipients. The expectations regarding the timing of specific behaviors can be differentiated into 2 categories: situation-independent and situation-dependent behaviors.

#### Interaction Skills and Behavior

Two core skills were identified as being essential for both health care professionals and patients: the capacity to provide structural support and the capacity to provide emotional support. These abilities correspond to the dimensions of interpersonal social perception, namely competence and warmth. An agent who exhibits these 2 abilities through appropriate behavior at the appropriate time could serve as a valuable addition to outpatient aftercare. For each subcluster and appropriate timing, the results provide an extensive list of suggestions for appropriate behavior.

#### Interaction Between Assistant and Health Professional

The results demonstrate the potential for therapists to obtain otherwise unavailable information regarding patients’ health status, as well as the feasibility of an emergency system in the event of suicidal risk. These innovations have the potential to address existing gaps in aftercare, enhance safety during this critical period, reduce the burden on health care professionals, and, by providing an objective measure of the patient’s health status, facilitate health care professional’s decision-making.

#### Summary of Conclusions

In summary, our findings offer a promising framework for the development of a VTA to support patients with depression during aftercare and support the work of health care professionals.

## Abbreviations

RQ: research question
SIA: socially interactive agent
VTA: virtual therapeutic assistant

## Data Availability

Following the recommendations for open research practices [50], questionnaires and experimenter instruction can be found on the OSF website [51]. The datasets generated or analyzed during this study are available from the corresponding author on reasonable request.
